# The origin of unidirectional charge separation in photosynthetic reaction centers: nonadiabatic quantum dynamics of exciton and charge in pigment–protein complexes†

**DOI:** 10.1039/d1sc01497h

**Published:** 2021-05-05

**Authors:** Hiroyuki Tamura, Keisuke Saito, Hiroshi Ishikita

**Affiliations:** Department of Applied Chemistry, The University of Tokyo 7-3-1 Hongo, Bunkyo-ku Tokyo 113-8654 Japan tamura@protein.rcast.u-tokyo.ac.jp; Research Center for Advanced Science and Technology, The University of Tokyo 4-6-1 Komaba, Meguro-ku Tokyo 153-8904 Japan

## Abstract

Exciton charge separation in photosynthetic reaction centers from purple bacteria (PbRC) and photosystem II (PSII) occurs exclusively along one of the two pseudo-symmetric branches (active branch) of pigment–protein complexes. The microscopic origin of unidirectional charge separation in photosynthesis remains controversial. Here we elucidate the essential factors leading to unidirectional charge separation in PbRC and PSII, using nonadiabatic quantum dynamics calculations in conjunction with time-dependent density functional theory (TDDFT) with the quantum mechanics/molecular mechanics/polarizable continuum model (QM/MM/PCM) method. This approach accounts for energetics, electronic coupling, and vibronic coupling of the pigment excited states under electrostatic interactions and polarization of whole protein environments. The calculated time constants of charge separation along the active branches of PbRC and PSII are similar to those observed in time-resolved spectroscopic experiments. In PbRC, Tyr-M210 near the accessary bacteriochlorophyll reduces the energy of the intermediate state and drastically accelerates charge separation overcoming the electron–hole interaction. Remarkably, even though both the active and inactive branches in PSII can accept excitons from light-harvesting complexes, charge separation in the inactive branch is prevented by a weak electronic coupling due to symmetry-breaking of the chlorophyll configurations. The exciton in the inactive branch in PSII can be transferred to the active branch *via* direct and indirect pathways. Subsequently, the ultrafast electron transfer to pheophytin in the active branch prevents exciton back transfer to the inactive branch, thereby achieving unidirectional charge separation.

## Abstract 2

Essential factors leading to unidirectional charge separation in photosynthetic reaction centers are clarified *via* nonadiabatic quantum dynamics calculations.

## Graphical abstract

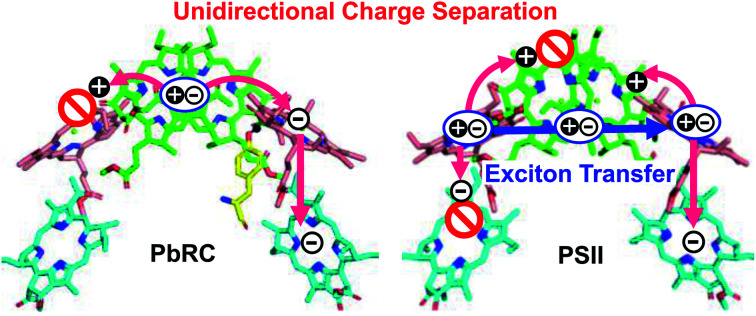

## 1. Introduction

Light reactions of photosynthesis achieve an extremely high internal quantum efficiency from photoabsorption to separated electrons and holes^1^ through ingeniously regulated pathways of energy and charge transfers in pigment–protein complexes. Light-harvesting (antenna) complexes, which contain a number of pigments, absorb a photon to create an electronically excited state characterized as a bound electron–hole pair, *i.e.* exciton.^1–7^ Exciton charge separation necessitates a sufficient potential difference between the donor and acceptor of electrons for overcoming the electron–hole Coulomb binding energy.

**Fig. 1:**
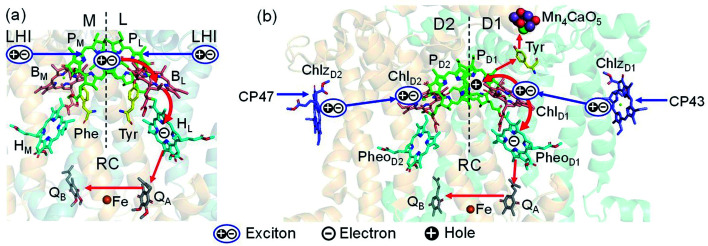
Exciton transfer^5–7,9,10^ and charge separation^11^ pathways in (a) PbRC and (b) PSII. Dashed lines indicate the rotation axis of pseudo-*C*_2_ symmetry. Blue and red arrows indicate exciton and charge transfer pathways, respectively. Phe and Tyr denote phenylalanine and tyrosine.

Photosystem II (PSII) consists of core antenna complexes (CP43 and CP47) and a reaction center (RC).^1–4^ Chlorophyll *a* (Chl) molecules in CP43 and CP47 mediate exciton transfers to the RC consisting of Chls (P_D1_, P_D2_, Chl_D1_, and Chl_D2_), pheophytin *a* (Pheo_D1_ and Pheo_D2_), and plastoquinone (Q_A_ and Q_B_) (Fig. 1).^8–38^ Charge separation occurs in the RC, where the electron reduces plastoquinone and the hole eventually oxidizes water at the Mn_4_CaO_5_ cluster.^11–13^ Similarly, bacteriochlorophyll *a* (BChl) molecules in the light harvesting complex I (LHI) from purple bacteria, *Rhodobacter sphaeroides*, transfer an exciton to the RC (PbRC) consisting of BChl (P_L_, P_M_, B_L_, and B_M_), bacteriopheophytin *a* (BPheo, H_L_ and H_M_), and ubiquinone (Q_A_ and Q_B_) (Fig. 1).^11^

Charge separation in PSII and PbRC occurs only along the active branch of the pseudo-*C*_2_ symmetric pigment–protein complexes, *i.e.*, D1- and L-branches, respectively (Fig. 1).^11^ The D2- and M-branches are referred to as inactive branches. PSII and PbRC may have been evolved from a common ancestor and classified as type II RCs.^11–13^ In type II RCs, Q_B_ in the inactive branch accepts an electron from Q_A_ in the active branch while it does not directly accept an electron from (B) Pheo in the inactive branch (Fig. 1).

In PbRC, the strong electronic coupling between the special pair BChls, P_L_ and P_M_, leads to stabilization of the delocalized exciton, (P_L_P_M_)*.^11,34^ The P_L_P_M_ can accept an exciton from LHI, which absorbs a near infrared photon.^5–7^ Time-resolved spectroscopic measurements indicated that the excited electron in (P_L_P_M_)* is transferred to H_L_*via* B_L_ along the L-branch on a time scale of a few ps.^39–50^ Despite the pseudo-*C*_2_ symmetric cofactor arrangement, the difference in the amino acid sequences between the L- and M-branches leads to the difference in the redox potentials of the pigments *via* electrostatic interactions and polarization.^51–53^

A previous study using time-dependent density functional theory (TDDFT) with the quantum mechanics/molecular mechanics/polarizable continuum model (QM/MM/PCM) method indicated that the intermediate states of charge separation along the L- and M-branches, *i.e.*, [P_L_P_M_]˙^+^B_L_˙^−^ and [P_L_P_M_]˙^+^B_M_˙^−^, are lower and higher in energy than that of (P_L_P_M_)*, respectively.^34^

In contrast to PbRC, the excitation energies of P_D1_ and P_D2_ in PSII are higher than those of Chl_D1_ and Chl_D2_,^9,34^ where the exciton tends to be localized on a single pigment owing to a weak excitonic coupling. Charge separation in PSII creates a hole localized on P_D1_˙^+^, which is the nearest pigment to the Mn_4_CaO_5_ cluster located on the D1 side.^54–57^ The localized nature of a hole on P_D1_˙^+^ is important for PSII to keep a high oxidation potential.^58^

In PSII, CP43 and CP47 transfer an exciton to the RC, presumably, *via* the peripheral Chls on the D1 (Chlz_D1_) and D2 (Chlz_D2_) sides.^3,9,10^ Time-resolved spectroscopic measurements on PSII suggested that the primary electron transfer occurs from an exciton on 
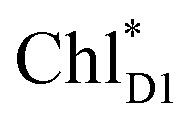
 to Pheo_D1_ on a time scale of a few hundred fs.^14,22^ The hole on Chl_D1_˙^+^ is, in turn, transferred to P_D1_ on a time scale of a few ps.^14^

Because the potential for electron transfer is energetically downhill along both the D1- and D2-branches toward Pheo_D1_ and Pheo_D2_, respectively,^33,34^ the energetics alone cannot explain unidirectional charge separation in PSII. Given that both Chl_D1_ and Chl_D2_ can accept an exciton from the core antenna complexes, the mechanism that leads to charge separation exclusively along the D1-branch is of particular interest. The charge separation pathways in pigment–protein complexes can be determined by various factors including energetics, electronic coupling, vibronic coupling, and quantum effects.^2–4,18–21,31,44–46^

In this study, we address the long-standing open question as to how PbRC and PSII achieve unidirectional charge separation exclusively along the active branch, by means of nonadiabatic quantum dynamics calculations^59–62^ parametrized on the basis of TDDFT in the framework of the QM/MM/PCM method.^63,64^ First, we show that the experimentally observed kinetics of charge separation along the active branches of PbRC and PSII are fairly well reproduced by nonadiabatic quantum dynamics calculations, which is based on the energetics and electronic coupling of the pigments, accounting for electrostatic interactions and polarization of whole protein environments from the X-ray crystal structures. On this basis, we clarify the essential factors which regulate the charge separation pathways in the reaction centers.

## 2. Methods

The energetics and electronic couplings in PbRC and PSII are analyzed by means of the polarizable QM/MM/PCM method, using the QuanPol code^63^ implemented in the GAMESS code.^65^ The electronic states in the QM regions are calculated using DFT and TDDFT with the CAMB3LYP functional^66^ with the range separation parameter *μ* of 0.14, *α* of 0.19, and *β* of 0.46, which is well suited for the present systems including charge separated states.^34^ The quantitative values of excitation energies may depend on functionals and parameters.^32,38,67^ The 6-31G(d) basis set is used for all the QM calculations.

The QM region comprises pigments, ligands, hydrogen bonded water, and residues which interact directly with pigments as detailed in a previous report.^34^ A polarizable amber-02 force field^68^ is applied for proteins in the MM region, where induced dipoles of the MM atoms are taken into account to reproduce the dielectric screening. The PCM with a dielectric constant of 80 is applied to reproduce the polarization of water, which surrounds the proteins and fills the cavities. The PCM in the QuanPol code is based on a conductor-like screening model,^63,64^ where the polarization points are put on spheres of radius 3.0 Å from the atom positions.^34^ All atoms from the X-ray crystal structures are explicitly considered, where each MM atom contains an induced dipole in addition to the permanent charge. The induced dipole of each MM atom is determined iteratively together with the self-consistent field calculation of electronic states, considering the electrostatic interactions with the electrons and nuclei in the QM region as well as the permanent charges and induced dipoles of other MM atoms.^63^ The molecular orbital levels of the cofactors calculated using QM/MM reproduce the redox potential values calculated solving the Poisson–Boltzmann equation.^33,34,57,69^ While the dielectric constant for the membrane region may be lower than 80 (*e.g.* 20),^70^ a small dielectric constant makes the electrostatic interactions with the charged groups in the membrane-extrinsic region overestimated for membrane proteins. The optimal values for the dielectric constant depend on the protein model used.^71,72^ The dielectric constant of 80 for the bulk region appears to be optimal for the present models, as suggested previously.^33,34^

The atomic coordinates of PSII and PbRC are obtained from the X-ray crystal structures from *Thermosynechococcus vulcanus* at 1.9 Å resolution (PDB code, 3ARC)^73^ and from *Rhodobacter sphaeroides* at 2.01 Å resolution (PDB code, 3I4D),^74^ respectively. The intramolecular reorganization energies of pigments are calculated through geometry optimization with QM/MM, where DFT with the CAMB3LYP functional plus Grimme's dispersion correction^75^ is used for the QM region. The atomic coordinates of the MM region are fixed to the X-ray crystal structures. The reorganization energy of the MM region is not taken into account.

The electronic coupling between excited states is evaluated on the basis of the diabatization scheme for TDDFT^76^ in the framework of the QM/MM/PCM method.^34,77^ The protocol of diabatization is summarized below.

(1) We prepare a set of reference wavefunctions, *Φ*_I_, that possess pure characters of the excited states such as an exciton on a single molecule (*i.e.*, Frenkel exciton) and charge separated states for decoupled molecules.

(2) We calculate adiabatic electronic states in the pigment–protein complexes using TDDFT-QM/MM/PCM.

(3) The diabatic wavefunctions are expressed as a linear combination of the adiabatic wavefunctions, *Ψ*_*J*_, by evaluating the overlap integrals between the reference and adiabatic wavefunctions:1
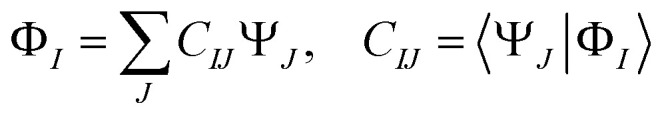


That is, the adiabatic states from the TDDFT-QM/MM/PCM calculations are considered as basis functions for expanding the diabatic states. We consider 10 adiabatic states for expanding the diabatic states. The diabatic coupling is then evaluated as follows:2
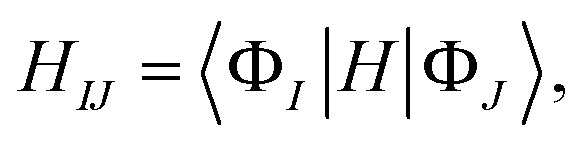
where *H* is the electronic Hamiltonian. The excitonic coupling in the present scheme includes both the Coulomb (Förster) and electron exchange (Dexter) contributions.^76^

For the nonadiabatic quantum dynamics calculations, we consider the following linear vibronic coupling Hamiltonian in the diabatic representation:3
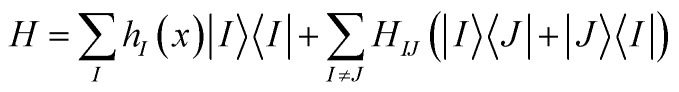
4
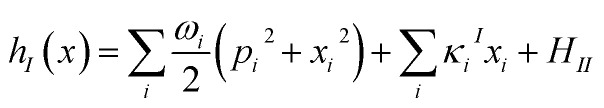


*H*_*IJ*_ is the diabatic coupling (electronic coupling) between the states *I* and *J*. *H*_*II*_ is the vertical excitation energy of the *I*th electronic states. *ω*_*i*_, *x*_*i*_, and *p*_*i*_ are the frequency, position, and momentum of the *i*th vibrational mode (harmonic oscillator) in the dimensionless coordinate. *κ*_*i*_^*I*^ is the vibronic coupling of the *i*th vibrational mode in the *I*th electronic state.

The exciton on the special pair, (P_L_P_M_)*, is considered for the initial conditions of the quantum dynamics calculations of charge separation in PbRC. For PSII, in addition to the exciton localized on 
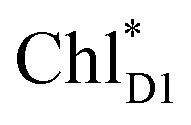
 in the D1-branch, 
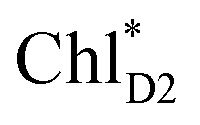
 in the D2-branch is also considered for the initial conditions of the quantum dynamics calculations of charge separation. For the exciton transfer between 
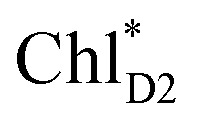
 and 
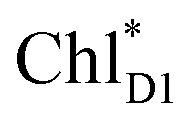
, the direct pathway and the indirect pathway *via*
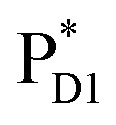
 and 
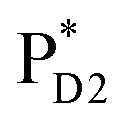
 are considered, where the quantum dynamics calculations account for the interference of the phase factors from several pathways. The initial vibrational wave packet is put on the Franck–Condon region of the initial electronic state.

*ω*_*i*_ and *κ*_*i*_^*I*^ in eqn (4), *i.e.*, spectral density, are determined on the basis of the normal mode analysis and the geometry optimization of the pigments using the QM/MM/PCM method, where the atomic displacements from the Franck–Condon region to the potential bottom on the respective electronic states are projected onto the normal modes. The present model explicitly considers the vibronic couplings of the pigments and axial ligands, which are relevant to the dynamics of charge separation on a time scale of a few ps, whereas slow vibrational modes from surrounding proteins are neglected. The vibrational modes are reduced to a limited number of effective modes which reproduce the short-time dynamics and the reorganization energy of the system (see ESI†).^60–62^ We consider 25 effective modes for each pigment, unless otherwise noted. For charge separation in PSII *via* indirect exciton transfer from 
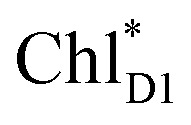
 to 
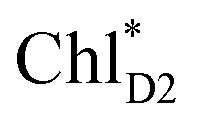
, 10 effective modes are considered for the respective intermediate states, 
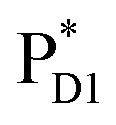
 and 
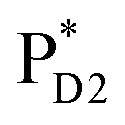
. The multi-configuration time-dependent Hartree (MCTDH) method^59^ is used for the nonadiabatic quantum dynamics calculations, which properly consider correlations among the nuclear degrees of freedom, the Franck–Condon factor of vibrational wavefunctions, and vibrational energy redistribution along with electronic state transitions.

For analyzing the time constants of the first (*τ*_1_) and second (*τ*_2_) charge transfers along the active branches, *τ*_1_ and *τ*_2_ in the following rate equations are determined *via* curve fitting against the populations of the exciton (*P*_EX_), and the first (*P*_CS1_) and second (*P*_CS2_) charge separated states in the quantum dynamics calculations:5
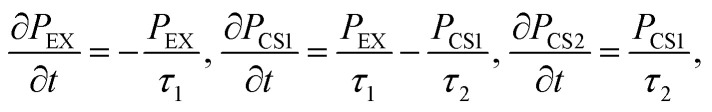
where the corresponding kinetic scheme is expressed as follows:6
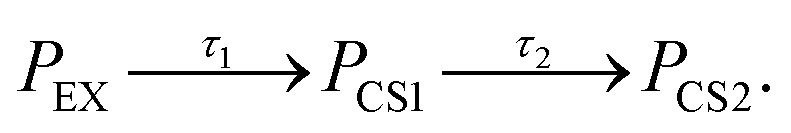


## 3. Results and discussion

### 3.1. Charge separation in PbRC

**Fig. 2:**
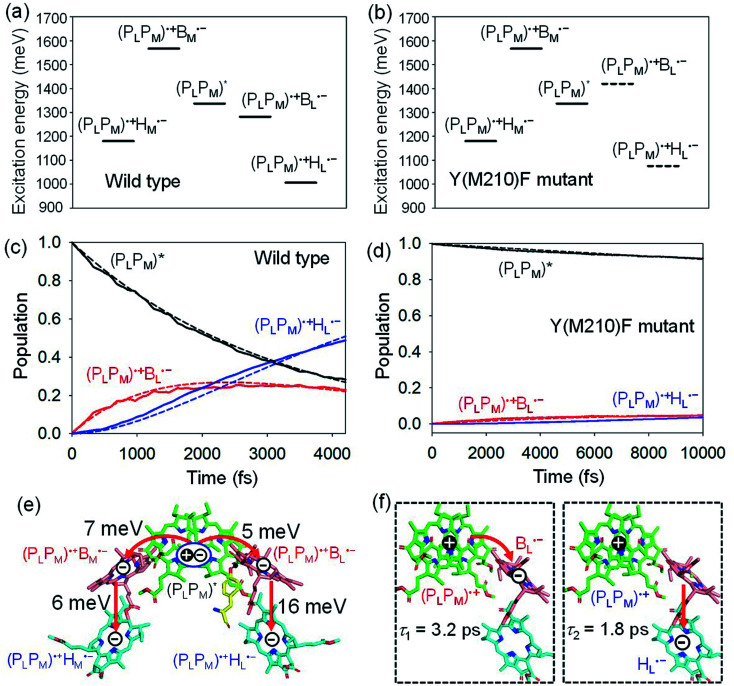
Bottom-to-bottom (adiabatic) excitation energies of the electronic states considering the intramolecular reorganization energies in (a) wild type and (b) Y(M210)F mutant PbRC. Dotted lines indicate the destabilized charge separated states in Y(M210)F mutant PbRC. Population of electronic states during quantum dynamics calculations of charge separation in (c) wild type and (d) Y(M210)F mutant PbRC, where the (P_L_P_M_)*, (P_L_P_M_)˙^+^B_L_˙^−^, and (P_L_P_M_)˙^+^H_L_˙^−^ states along the L-branch are considered. Dotted lines indicate curve fitting by using eqn (5). (e) Diagram of charge transfer pathways (red lines) with electronic coupling (meV). Tyr-M210 is shown in yellow. (f) Diagram of the electron and hole locations in the (P_L_P_M_)˙^+^B_L_˙^−^ and (P_L_P_M_)˙^+^H_L_˙^−^ states with *τ*_1_ and *τ*_2_ (ps) for wild type PbRC (∼5 ps in total). *τ*_1_ and *τ*_2_ for the mutant PbRC are 110 and 8 ps, respectively (∼118 ps in total).

(P_L_P_M_) in PbRC can be regarded as a single molecular site owing to the strong electronic coupling.^34^ The electron transfers from (P_L_P_M_)* to (P_L_P_M_)˙^+^B_L_˙^−^ and (P_L_P_M_)˙^+^B_M_˙^−^ are exothermic (downhill) and endothermic (uphill), respectively (Fig. 2a).^34^ As a benchmark, we first compare the calculated time constants of charge separation along the L-branch with the corresponding experimental values. The quantum dynamics calculations indicate that (P_L_P_M_)* initially transfers the excited electron to B_L_ on a time scale of *τ*_1_ ≈ 3.2 ps. B_L_˙^−^, in turn, transfers the electron to H_L_ on a time scale of *τ*_2_ ≈ 1.8 ps (Fig. 2c and f). A similar order of time constants was observed in time-resolved spectroscopic measurements on charge separation in PbRC (*τ*_1_ = 3.5 ± 0.4 ps and *τ*_2_ = 1.2 ± 0.3 ps).^41^

The electronic coupling of the B_L_˙^−^ → H_L_˙^−^ transfer (16 meV) is stronger than that of the (P_L_P_M_)* → (P_L_P_M_)˙^+^B_L_˙^−^ transfer (5 meV) (Fig. 2e). Thus, the population of the intermediate (P_L_P_M_)˙^+^B_L_˙^−^ state is kept small (Fig. 2c). The fast electron transfer from B_L_˙^−^ to H_L_ is advantageous for preventing charge recombination, because (P_L_P_M_)˙^+^H_L_˙^−^ is difficult to decay to the ground state owing to a negligibly small orbital overlap between (P_L_P_M_)˙^+^ and H_L_˙^−^. Charge separation along the M-branch is negligibly slow, because the intermediate (P_L_P_M_)˙^+^B_M_˙^−^ state is substantially higher in energy than (P_L_P_M_)*, even though (P_L_P_M_)˙^+^H_M_˙^−^ is lower in energy than (P_L_P_M_)* (Fig. 2a).

The previous time-resolved spectroscopic measurements of mutant PbRC suggested that some specific residues especially contribute to unidirectional charge separation.^47–50,52^ We have extensively analyzed the contribution of each residue to the potential shift on the pigments one by one, and concluded that Tyr-M210 near B_L_ has the largest contribution to the stabilization of B_L_˙^−^,^33,34^ where Phe-L181 is located at the counterpart position near B_M_.

To verify the essential role of Tyr-M210, we consider the mutation of Tyr-M210 to phenylalanine, Y(M210)F, and investigate charge separation in the mutant PbRC by means of quantum dynamics calculations. The present TDDFT-QM/MM/PCM calculations indicate that the Y(M210)F mutation, in which the hydroxyl group is replaced with hydrogen, makes the intermediate (P_L_P_M_)˙^+^B_L_˙^−^ state energetically uphill with respect to (P_L_P_M_)*, even though the final (P_L_P_M_)˙^+^H_L_˙^−^ state remains downhill (Fig. 2b). The quantum dynamics calculation indicates that the destabilization of the intermediate (P_L_P_M_)˙^+^B_L_˙^−^ state drastically slows charge separation along the L-branch through the superexchange mechanism (Fig. 2d). This trend is qualitatively consistent with the experimental observations for the mutant PbRC,^47–50,52^ where the calculated time constant (∼118 ps) is quantitatively larger than the experimental values (∼16 ps).^47^ Here, only the local geometry of Phe-M210 was optimized in QM/MM, fixing surrounding proteins at the original geometry of wild type, although the mutation may also affect the surrounding geometry. Overall, the present analysis highlights the impact of the electrostatic interaction of Tyr-M210 on the efficient charge separation along the L-branch.

### 3.2. Charge separation in PSII

**Fig. 3:**
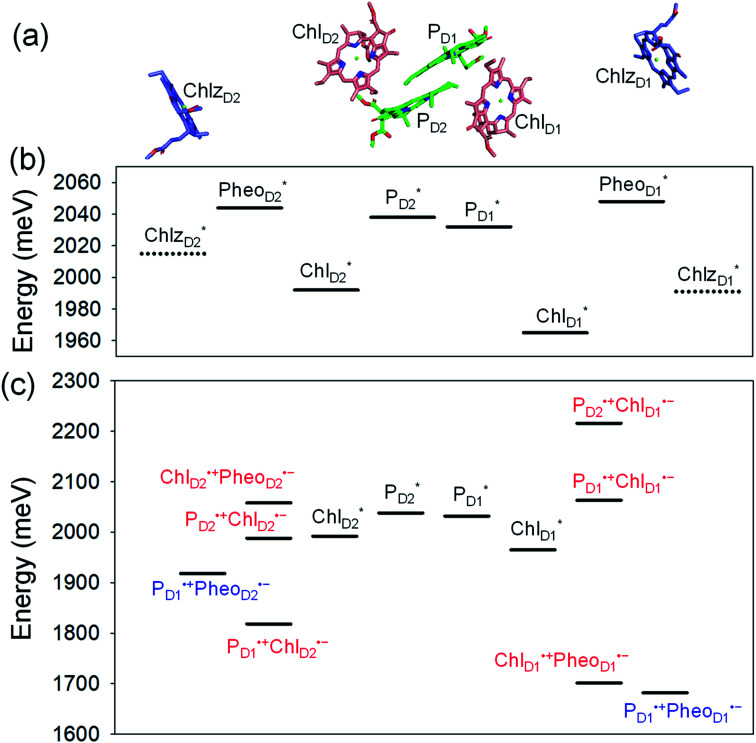
(a) Arrangement of Chl molecules in PSII. Calculated bottom-to-bottom (adiabatic) excitation energies considering the intramolecular reorganization energies of the (b) exciton and (c) exciton and charge separated states in PSII. The intermediate and final charge separated states are indicated in red and blue, respectively.

In PSII, Chl_D1_ and Chl_D2_ are supposed to accept an exciton from CP43 and CP47 *via*
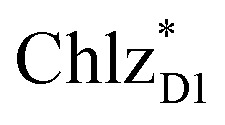
 and 
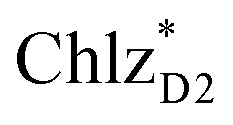
, respectively (Fig. 3a).^9,10^ The present calculations indicate that the bottom-to-bottom excitation energy of 
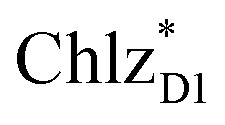
 (1991 meV) lies between those of 
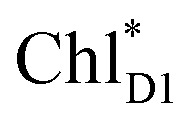
 (1965 meV) and 
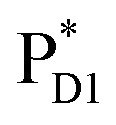
 (2032 meV, Fig. 3b). Similarly, the 
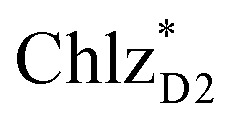
 energy (2015 meV) lies between those of 
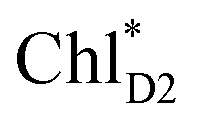
 (1992 meV) and 
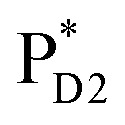
 (2038 meV, Fig. 3b). Thus, 
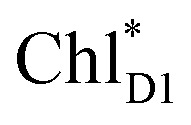
 and 
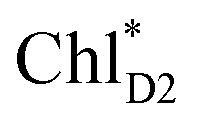
 can accept an exciton from 
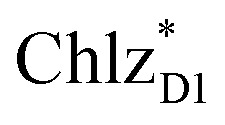
 and 
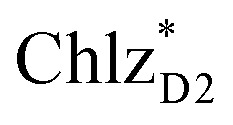
, respectively, in terms of energetics.

The absolute values of the calculated excitonic couplings in PSII are in the range of 7 to 15 meV (Table 1). The lowest and second lowest excitons obtained by diagonalizing the coupling matrix are localized on 
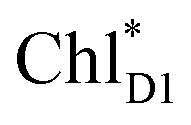
 and 
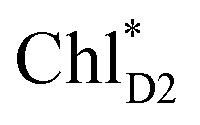
, respectively (Fig. S2†), which can be regarded as Frenkel excitons. The quantitative values of the exciton energies in PSII calculated using TDDFT-QM/MM/PCM with the CAMB3LYP functional tend to be blue-shifted as compared to the experimental values,^25–27^ where the calculated lowest exciton energy of 632 nm is blue-shifted as compared to the experimental value of 680 nm.^25–27^

**Table 1:** Electronic coupling in PSII (meV)

	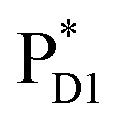	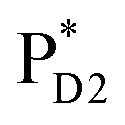	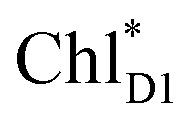	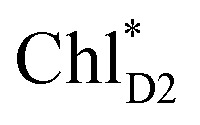	Chl_D1_˙^+^Pheo_D1_˙^−^
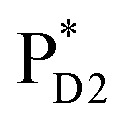	−10.1				
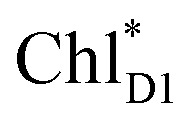	7.4	−13.6			
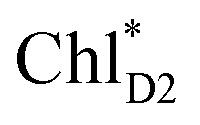	−14.4	7.0	−1.8		
Chl_D1_˙^+^Pheo_D1_˙^−^			−21.6		
P_D1_˙^+^Pheo_D1_˙^−^					6.4
P_D1_˙^+^Chl_D1_˙^−^	−6.5		5.4		
P_D1_˙^+^Chl_D2_˙^−^	0.7			−0.3	
P_D2_˙^+^Chl_D1_˙^−^		−0.5	−5.4		

**Fig. 4:**
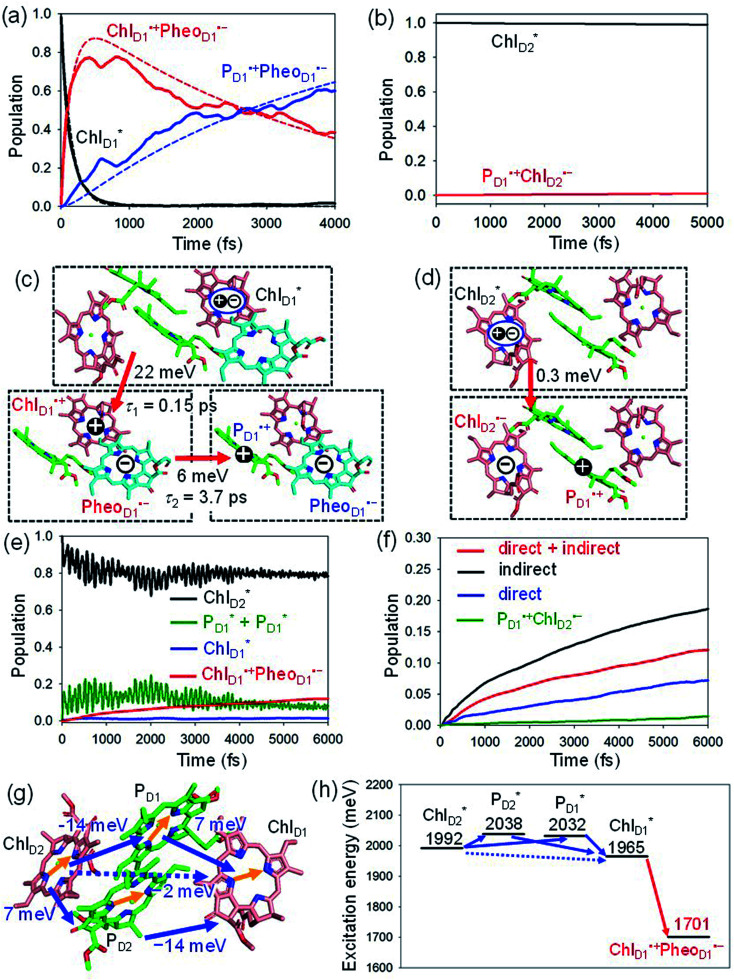
Population of excited states during quantum dynamics calculations of charge separation in PSII: (a) 
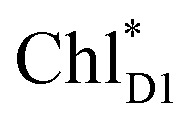
, Chl_D1_˙^+^Pheo_D1_˙^−^, and P_D1_˙^+^Pheo_D1_˙^−^ along the D1-branch and (b) from 
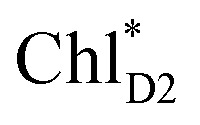
 to P_D1_˙^+^Chl_D2_˙^−^. Dotted lines indicate curve fitting by using eqn (5). Diagram of charge separation with the absolute value of electronic coupling (meV): (c) from 
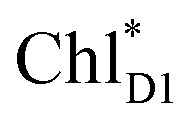
 and (d) from 
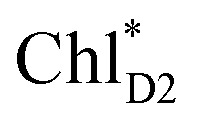
. Population of excited states during quantum dynamics calculations from 
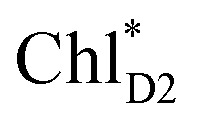
: (e) simultaneously considering indirect 
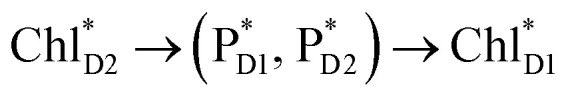
 → Chl_D1_˙^+^Pheo_D1_˙^−^ (*τ* ≈ 50 ps) and direct 
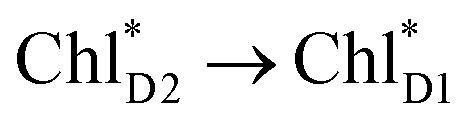
 → Chl_D1_˙^+^Pheo_D1_˙^−^ pathways, and (f) Chl_D1_˙^+^Pheo_D1_˙^−^ population considering only the indirect (black) or the direct (blue) pathway. The P_D1_˙^+^Chl_D2_˙^−^ population (green) is shown again for comparison. (g) Exciton transfer pathways (blue arrows) with the excitonic coupling (meV). Orange arrows indicate the direction of transition dipole moment. Solid and dotted arrows indicate indirect and direct exciton transfers, respectively. (h) Exciton transfer and charge separation pathways with the excitation energy.

We analyze charge separation from an exciton on 
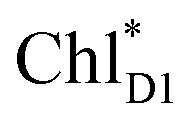
 by means of quantum dynamics calculations. The initial electron transfer from 
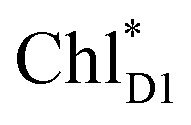
 to Chl_D1_˙^+^Pheo_D1_˙^−^ occurs on an ultrafast time scale (*τ*_1_ ≈ 0.15 ps) (Fig. 4a and c) owing to a strong electronic coupling (∼22 meV, Fig. 4c and Table 1). The subsequent hole transfer to P_D1_˙^+^Pheo_D1_˙^−^ occurs on a time scale of *τ*_2_ ≈ 3.7 ps (Fig. 4a and c). Thus, once 
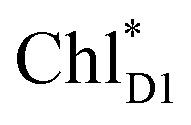
 accepts an exciton, charge separation occurs efficiently along the D1-branch. Similar time constants of charge separation in PSII were observed in the time-resolved spectroscopy measurements.^14,22^ Another charge separation pathway, 
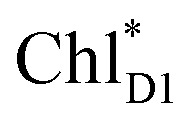
 → P_D1_˙^+^Chl_D1_˙^−^, is endothermic (Fig. 3c) and thus cannot compete with 
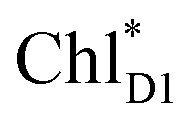
 → Chl_D1_˙^+^Pheo_D1_˙^−^. Although other charge separation pathways from an exciton on 
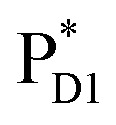
 and 
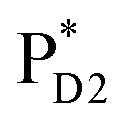
 were also proposed,^23,24^ the quantum dynamical analysis for these pathways is beyond the scope of the present study. Overall, we can conclude that charge separation along the D1-branch proceeds *via* two-step 
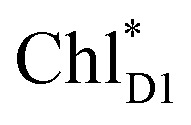
 → Chl_D1_˙^+^Pheo_D1_˙^−^ and Chl_D1_˙^+^ → P_D1_˙^+^ transfers, considering the quantum dynamical analysis based on the energetics and electronic couplings from the QM/MM/PCM method.

The electronic coupling of the 
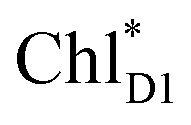
 → Chl_D1_˙^+^Pheo_D1_˙^−^ transfer (∼22 meV) is stronger than that of the Chl_D1_˙^+^ → P_D1_˙^+^ transfer (∼6 meV, Fig. 4c and Table 1). The strong electronic coupling between the accessary Chl/BChl and the Pheo/BPheo is a common feature of PSII/PbRC. Nevertheless, the B_L_˙^−^ → H_L_˙^−^ electron transfer (∼1.8 ps) in PbRC is slower than the 
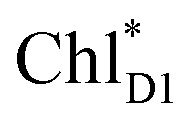
 → Chl_D1_˙^+^Pheo_D1_˙^−^ transfer (∼0.15 ps) in PSII, because the population of the (P_L_P_M_)˙^+^B_L_˙^−^ intermediate state in PbRC is kept small.

Because Chl_D2_ can also accept an exciton form CP47 on the D2 side,^3,9,10^ the question arises as to how the exciton on 
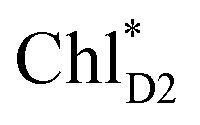
 eventually undergoes charge separation in the D1-branch. To analyze charge separation mechanisms from an exciton in the D2-branch, we carried out quantum dynamics calculations considering the initial exciton localized on 
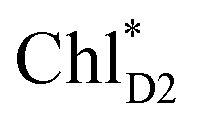
. The charge separated state in the D2-branch, P_D1_˙^+^Pheo_D2_˙^−^, is less stable than that in the D1-branch, P_D1_˙^+^Pheo_D1_˙^−^ (Fig. 3c), owing mainly to a difference in the potentials between Pheo_D1_˙^−^ and Pheo_D2_˙^−^.^33,34,36^

**Fig. 5:**
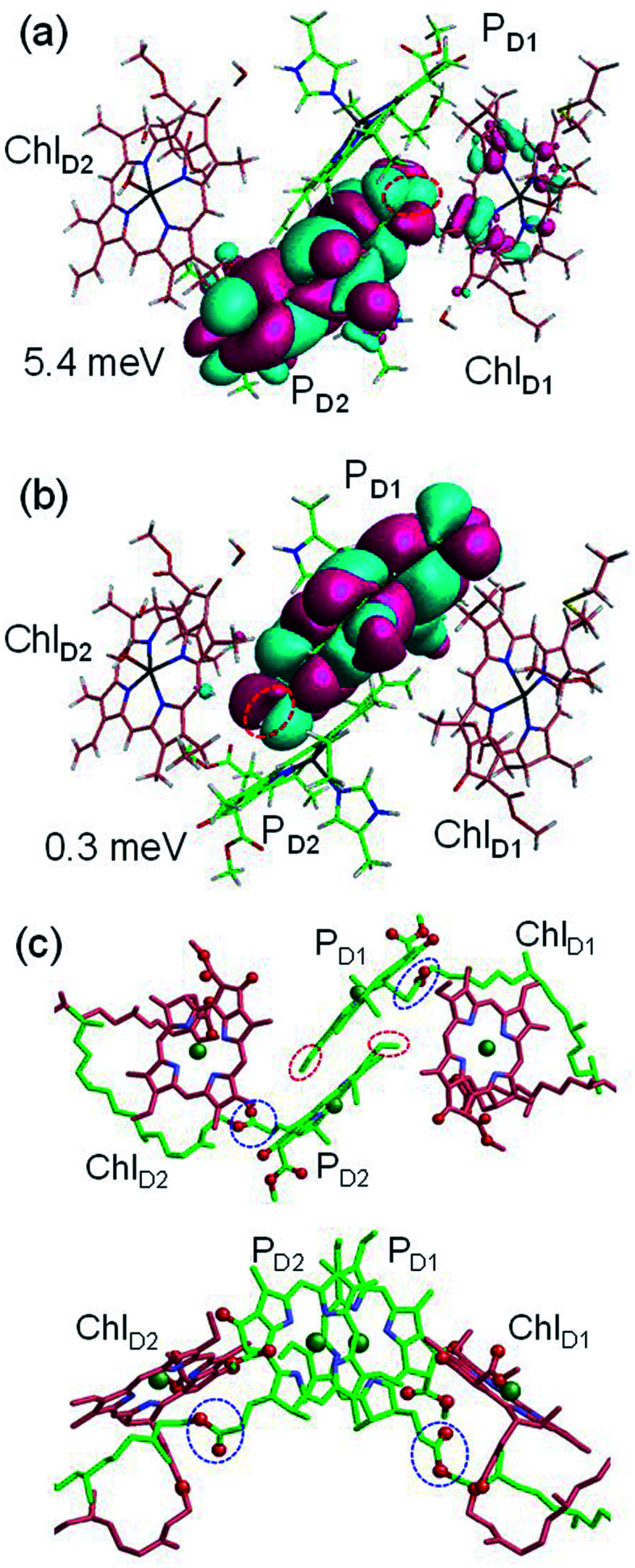
Equilibrium geometry and the highest occupied molecular orbital (HOMO) of the (a) P_D2_Chl_D1_ dimer and (b) P_D1_Chl_D2_ dimer. Red circles indicate the vinyl groups. Electronic coupling (meV) between 
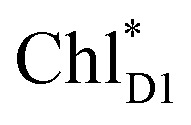
 and P_D2_˙^+^Chl_D1_˙^−^ and that between 
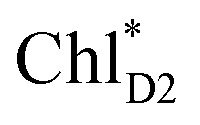
 and P_D1_˙^+^Chl_D2_˙^−^ are shown. (c) Configuration of the vinyl (red circles) and phytol (blue circles) groups of P_D1_ and P_D2_.

**Fig. 6:**
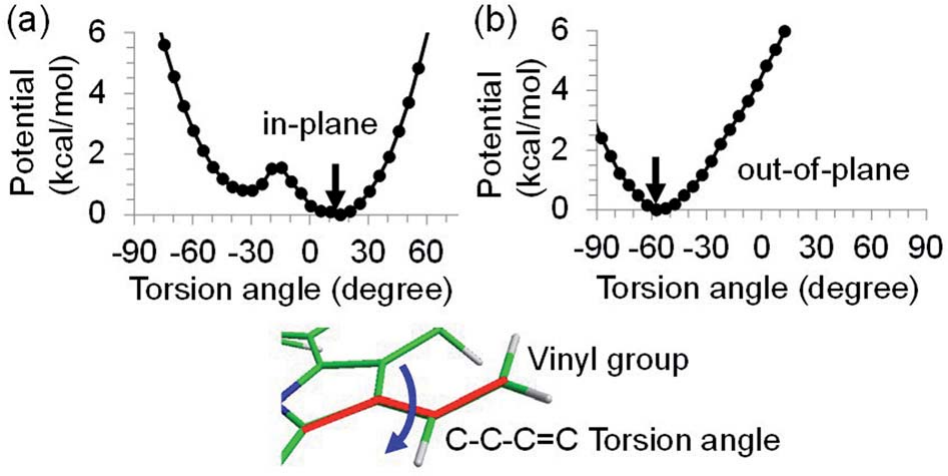
Potential energy curve as a function of the C–C–C=C torsion angle (degree) of the vinyl group of (a) P_D1_ and (b) P_D2_ in PSII, where the geometry of chlorophyll is optimized by QM/MM, fixing the torsion angle. The vinyl group is in the same plane as the chlorin ring at 0 degree. The black arrows indicate the equilibrium angle.

The most stable charge separated state in the D2-branch is P_D1_˙^+^Chl_D2_˙^−^ (Fig. 3c). However, PSII can avoid charge separation from 
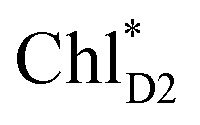
 to P_D1_˙^+^Chl_D2_˙^−^ (Fig. 4b) because of a weak electronic coupling (∼0.3 meV, Fig. 4d and Table 1), which is significantly weaker than that between 
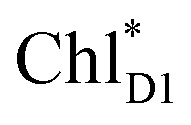
 and P_D2_˙^+^Chl_D1_˙^−^ (∼5.4 meV) on the counterpart side (Fig. 5a and Table 1). The difference originates from the difference in the vinyl-group orientation between P_D1_ and P_D2_ (Fig. 5). The vinyl group is rather in plane for P_D1_ and out of plane for P_D2_ (Fig. 6).^57^ The present results indicate that the in-plane P_D1_ vinyl group interferes with the π–π interaction between P_D1_ and Chl_D2_ (Fig. 5b), thereby preventing the charge transfer to form P_D1_˙^+^Chl_D2_˙^−^.

The out-of-plane orientation of the P_D2_ vinyl group is caused by the relatively large steric hindrance from the P_D1_ phytol chain as compared with that between the P_D1_ vinyl group and the P_D2_ phytol chain (Fig. 5c and 6). The potential curves calculated using QM/MM indicate that the rotations of the P_D1_ and P_D2_ vinyl groups are hindered in the protein environments (Fig. 6 and S1†). Umena *et al.* reported that the conformations of vinyl groups were determined unambiguously from the corresponding electron density distributions and most of the vinyl groups are located in or near the same plane of the chlorine ring,^73^ which suggests that the out-of-plane vinyl orientation for P_D2_ is exceptional. The same conformations of the P_D1_ and P_D2_ vinyl groups have also been observed in the X-ray free electron laser (XFEL) structure.^78^ Thus, the observed vinyl orientations of P_D1_ and P_D2_ are considered to be robust in the protein environments. The phytol chains of P_D1_ and P_D2_ are less flexible due to the presence of the highly packed protein environment of D1/D2/CP43/CP47, as compared to those exposed to the protein surface in antenna proteins (*e.g.*, LH1 and the Fenna–Matthews–Olson protein). Umena *et al.* also confirmed that all of the C8 and C13 positions in the phytol chains have a (*R*,*R*) configuration as indicated by the low *B*-factor values.^73^ Notably, the difference in the phytol-chain conformation also contributes to the asymmetric hole distribution on P_D1_ and P_D2_, *i.e.*, P_D1_˙^+^ > P_D2_˙^+^.^57^ These results suggest that the symmetry-breaking of the P_D1_P_D2_ geometry not only increases the P_D1_˙^+^ population, which facilitates water oxidation at the Mn_4_CaO_5_ moiety on the D1 side, but also prevents charge separation along the D2-branch *via* a weak electronic coupling between 
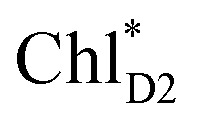
 and P_D1_˙^+^Chl_D2_˙^−^.

The quantum dynamics calculations indicate that the exciton on 
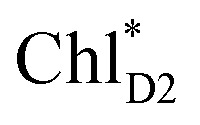
 can be transferred to Chl_D1_*via* the direct pathway and the indirect pathway mediated by 
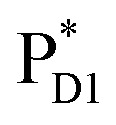
 and 
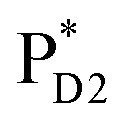
 owing to adequately strong excitonic couplings (Table 1 and Fig. 4g) and small energy differences (Fig. 4h). Note that among the residues near Chl_D1_ and Chl_D2_, D1-Met172 adjacent to Chl_D1_ contributes to the difference in excitation energy between 
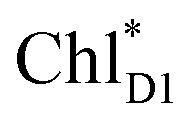
 and 
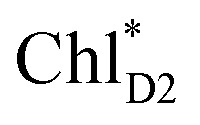
.^34^ Previous calculations by Sirohiwal *et al.*^38^ using other DFT functionals and an equation-of-motion coupled cluster method also indicated that Chl_D1_ exhibits the lowest excitation energy in the protein environment of PSII. Once the exciton is transferred to Chl_D1_, subsequent charge separation to Chl_D1_˙^+^Pheo_D1_˙^−^ occurs rapidly. The present analysis indicates that an overall time scale of charge separation from 
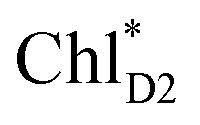
 to Chl_D1_˙^+^Pheo_D1_˙^−^ is in the order of a few tens ps (Fig. 4e and f), where exponential fitting indicates a *τ* of ∼50 ps. It is highly likely that the exciton on 
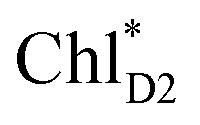
 can eventually undergo charge separation in the D1-branch without charge separation in the D2-branch. Even though the energy difference between 
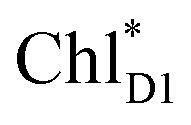
 and 
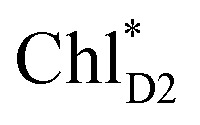
 is small (Fig. 4h), the ultrafast charge separation from 
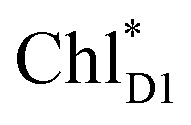
 to Chl_D1_˙^+^Pheo_D1_˙^−^ prevents exciton back transfer to Chl_D2_, enhancing the robustness of unidirectional charge separation along the D1-branch. The charge separation pathway *via* the exciton transfer from the D2- to D1-branches may correspond to the delayed component observed in the time-resolved spectroscopic measurements apart from the ultrafast 
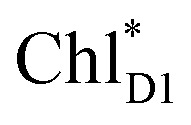
 → Chl_D1_˙^+^Pheo_D1_˙^−^ charge separation within the D1-branch.^14^

The direct excitonic coupling between 
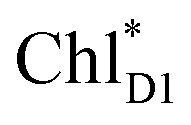
 and 
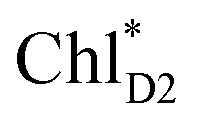
 is relatively weak (∼2 meV) owing to the long distance (∼20 Å) as compared with the coupling between neighboring Chls, *i.e.*, 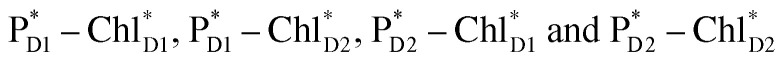 pairs (Fig. 4g and Table 1). Consequently, charge separation considering only the direct 
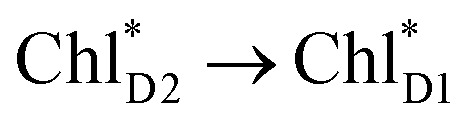
 → Chl_D1_˙^+^Pheo_D1_˙^−^ pathway is slower than charge separation considering only the indirect 
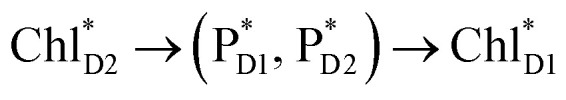
 → Chl_D1_˙^+^Pheo_D1_˙^−^ pathway in the quantum dynamics calculations (Fig. 4f). The excitonic coupling between 
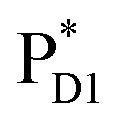
 and 
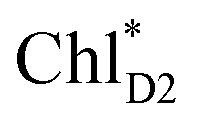
 (−14 meV) is stronger than that between 
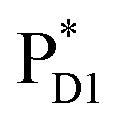
 and 
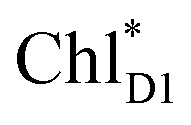
 (7 meV, Table 1). The former and latter are characterized as J- and H-aggregates (minus and plus signs), respectively, considering the directions of the transition dipole moments (Fig. 4g). The excitonic coupling is relatively insensitive to the orbital overlap as compared with the case of the charge transfer coupling. The direct and indirect 
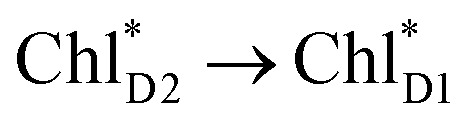
 exciton transfers exhibit the destructive interference of the quantum phase factor, which is dictated by the signs of excitonic couplings, *i.e.*, relative orientation of the transition dipole moments. Consequently, the exciton transfer rate considering all pathways is slower than the rate considering only the indirect pathway (Fig. 4f). Thus, in terms of the phase factor, the configuration of Chls in PSII is not necessarily optimal for accelerating the 
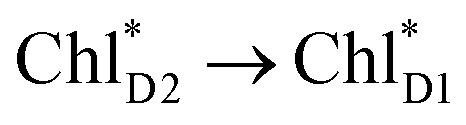
 transfer, while the configuration is optimal for charge separation along the D1-branch.

Overall, it can be concluded that the 
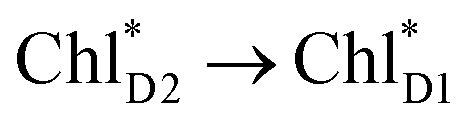
 exciton transfer followed by charge separation to Chl_D1_˙^+^Pheo_D1_˙^−^ in the D1-branch is overwhelmingly faster than charge separation in the D2-branch (Fig. 4f). The irreversible 
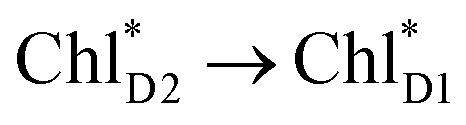
 exciton transfer allows PSII to utilize the excitation energy from both the CP43 and CP47 antenna complexes for charge separation in the active branch.

### 3.3. Role of Mn_4_CaO_5_ in the charge separation pathway in PSII

The localized electronic states on P_D1_ in PSII are advantageous to maintain a high oxidation potential for water splitting in contrast to the strongly coupled (P_L_P_M_)* and (P_L_P_M_)˙^+^ in PbRC.^34^ The hole on P_D1_˙^+^ is largely stabilized by acidic residues near the Mn_4_CaO_5_ cluster, namely D1-Asp61, D1-Glu189, and D1-Asp170.^34,57^ This may explain why the Mn_4_CaO_5_ cluster is located on the D1 side, because the electrostatic potential, which attracts a hole toward the D1 side, also enhances charge separation to P_D1_˙^+^Pheo_D1_˙^−^.^33,34,58^ Because the difference in the redox potential between P_D1_ and Chl_D1_ is small,^34^ P_D1_˙^+^Chl_D1_˙^−^ is substantially higher in energy than Chl_D1_˙^+^Pheo_D1_˙^−^ (Fig. 3c). Thus, the exciton funneling to 
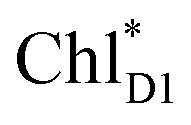
 rather than 
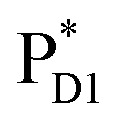
 is a reasonable design principle for efficient charge separation to use excitons from the antenna complexes in PSII.

## 4. Conclusion

Quantum dynamics calculations indicated that two-step (P_L_P_M_)* → (P_L_P_M_)˙^+^B_L_˙^−^ and B_L_˙^−^ → H_L_˙^−^ electron transfers occur on a time scale of ∼3.2 and ∼1.8 ps, respectively (Fig. 2c). The population of the intermediate (P_L_P_M_)˙^+^B_L_˙^−^ state is kept small, owing to a strong B_L_˙^−^ → H_L_˙^−^ coupling (∼16 meV, Fig. 2e). The rapid electron transfer to H_L_ is advantageous for preventing charge recombination, because the orbital overlap between (P_L_P_M_)˙^+^ and H_L_˙^−^ is negligibly small owing to a long molecular distance. The electrostatic interaction with the hydroxyl group of Tyr-M210 near B_L_ stabilizes the intermediate (P_L_P_M_)˙^+^B_L_˙^−^ state and accelerates charge separation along the L-branch, highlighting the essential role of Tyr-M210 in efficient unidirectional charge separation.

In PSII, both Chl_D1_ and Chl_D2_ can accept an exciton from CP43 and CP47, respectively. The 
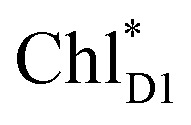
 → Chl_D1_˙^+^Pheo_D1_˙^−^ electron transfer occurs on an ultrafast time scale (∼0.15 ps), followed by the Chl_D1_˙^+^ → P_D1_˙^+^ hole transfer on a time scale of ∼3.7 ps (Fig. 4a), as suggested by time-resolved spectroscopic measurements.^14^ Charge separation in the D2-branch is unlikely to occur despite the relatively stable P_D1_˙^+^Chl_D2_˙^−^ state, because the in-plane P_D1_ vinyl group interferes with the π–π interaction between P_D1_ and Chl_D2_, thereby weakening the electronic coupling. The exciton on 
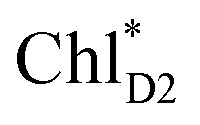
 can be transferred to Chl_D1_*via* the direct and indirect pathways. Subsequently, the ultrafast 
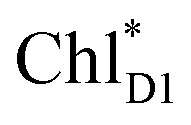
 → Chl_D1_˙^+^Pheo_D1_˙^−^ charge separation prevents exciton back transfer to Chl_D2_, thereby enhancing the robustness of unidirectional charge separation in the D1-branch. Thus, PSII efficiently utilizes excitons not only from CP43 (D1 side) but also from CP47 (D2 side) for charge separation in the D1-branch, which leads to electron transfer to Q_B_*via* Q_A_ and hole transfer to the Mn_4_CaO_5_ cluster on the D1 side.

## Author contributions

H. T. designed the research. H. T., K. S., and H. I. performed the research. H. T. wrote the main part of the manuscript. All the authors were involved in the discussion of the results and contributed to the final version of the manuscript.

## Conflicts of interest

There are no conflicts to declare.

## Supplementary Material

SC-012-D1SC01497H-s001

SC-012-D1SC01497H-s002

## References

[cit1] Wientjes E., van Amerongen H., Croce R. (2013). J. Phys. Chem. B.

[cit2] Scholes G. D., Fleming G. R., Olaya-Castro A., van Grondelle R. (2011). Nat. Chem..

[cit3] Kreisbeck C., Aspuru-Guzik A. (2016). Chem. Sci..

[cit4] Pan J., Gelzinis A., Chorošajev V., Vengris M., Senlik S. S., Shen J.-R., Valkunas L., Abramavicius D., Ogilvie J. P. (2017). Phys. Chem. Chem. Phys..

[cit5] van Grondelle R., Novoderezhkin V. I. (2006). Phys. Chem. Chem. Phys..

[cit6] Ma F., Yu L.-J., Wang-Otomo Z.-Y., van Grondelle R. (2016). Biochim. Biophys. Acta Bioenerg..

[cit7] Tan L.-M., Yu J., Kawakami T., Kobayashi M., Wang P., Wang-Otomo Z.-Y., Zhang J.-P. (2018). J. Phys. Chem. Lett..

[cit8] Germano M., Shkuropatov A. Y., Permentier H., de Wijn R., Hoff A. J., Shuvalov V. A., van Gorkom H. J. (2001). Biochemistry.

[cit9] Diner B. A., Rappaport F. (2002). Annu. Rev. Plant Biol..

[cit10] Vasil’ev S., Shen J.-R., Kamiya N., Bruce D. (2004). FEBS Lett..

[cit11] Cardona T., Sedoud A., Cox N., Rutherford A. W. (2012). Biochim. Biophys. Acta Bioenerg..

[cit12] Cardona T., Rutherford A. W. (2019). Trends Plant Sci..

[cit13] Cardona T., Murray J. W., Rutherford A. W. (2015). Mol. Biol. Evol..

[cit14] Groot M. L., Pawlowicz N. P., van Wilderen L. J. G. W., Breton J., van Stokkum I. H. M., van Grondelle R. (2005). Proc. Natl. Acad. Sci. U. S. A..

[cit15] Groot M.-L., van Mourik F., Eijckelhoff C., van Stokkum I. H. M., Dekker J. P., van Grondelle R. (1997). Proc. Natl. Acad. Sci. U. S. A..

[cit16] Holzwarth A. R., Müller M. G., Reus M., Nowaczyk M., Sander J., Rögner M. (2006). Proc. Natl. Acad. Sci. U. S. A..

[cit17] Hasegawa M., Nagashima H., Minobe R., Tachikawa T., Mino H., Kobori Y. (2017). J. Phys. Chem. Lett..

[cit18] Myers J. A., Lewis K. L. M., Fuller F. D., Tekavec P. F., Yocum C. F., Ogilvie J. P. (2010). J. Phys. Chem. Lett..

[cit19] Fuller F. D., Pan J., Gelzinis A., Butkus V., Senlik S. S., Wilcox D. E., Yocum C. F., Valkunas L., Abramavicius D., Ogilvie J. P. (2014). Nat. Chem..

[cit20] Romero E., Augulis R., Novoderezhkin V. I., Ferretti M., Thieme J., Zigmantas D., van Grondelle R. (2014). Nat. Phys..

[cit21] Romero E., Novoderezhkin V. I., van Grondelle R. (2017). Nature.

[cit22] Raszewski G., Renger T. (2008). J. Am. Chem. Soc..

[cit23] Romero E., van Stokkum I. H. M., Novoderezhkin V. I., Dekker J. P., van Grondelle R. (2010). Biochemistry.

[cit24] Novoderezhkin V. I., Romero E., Dekker J. P., van Grondelle R. (2011). ChemPhysChem.

[cit25] Raszewski G., Saenger W., Renger T. (2005). Biophys. J..

[cit26] Novoderezhkin V. I., Dekker J. P., van Grondelle R. (2007). Biophys. J..

[cit27] Acharya K., Neupane B., Zazubovich V., Sayre R. T., Picorel R., Seibert M., Jankowiak R. (2012). J. Phys. Chem. B.

[cit28] Raszewski G., Diner B. A., Schlodder E., Renger T. (2008). Biophys. J..

[cit29] Müh F., Plöckinger M., Renger T. (2017). J. Phys. Chem. Lett..

[cit30] Gelzinis A., Valkunas L., Fuller F. D., Ogilvie J. P., Mukamel S., Abramavicius D. (2013). New J. Phys..

[cit31] Fujihashi Y., Higashi M., Ishizaki A. (2018). J. Phys. Chem. Lett..

[cit32] Kavanagh M. A., Karlsson J. K. G., Colburn J. D., Barter L. M. C., Gould I. R. (2020). Proc. Natl. Acad. Sci. U. S. A..

[cit33] Kawashima K., Ishikita H. (2018). Chem. Sci..

[cit34] Tamura H., Saito K., Ishikita H. (2020). Proc. Natl. Acad. Sci. U. S. A..

[cit35] Mandal M., Kawashima K., Saito K., Ishikita H. (2020). J. Phys. Chem. Lett..

[cit36] Ishikita H., Biesiadka J., Loll B., Saenger W., Knapp E.-W. (2006). Angew. Chem., Int. Ed..

[cit37] Zhang L., Silva D.-A., Zhang H., Yue A., Yan Y., Huang X. (2014). Nat. Commun..

[cit38] Sirohiwal A., Neese F., Pantazis D. A. (2020). J. Am. Chem. Soc..

[cit39] Ziolek M., Pawlowicz N., Naskrecki R., Dobek A. (2005). J. Phys. Chem. B.

[cit40] Wang H., Lin S., Woodbury N. W. (2008). J. Phys. Chem. B.

[cit41] Lauterwasser C., Finkele U., Scheer H., Zinth W. (1991). Chem. Phys. Lett..

[cit42] Zinth W., Wachtveitl J. (2005). ChemPhysChem.

[cit43] Niedringhaus A., Policht V. R., Sechrist R., Konar A., Laible P. D., Bocian D. F., Holten D., Kirmaier C., Ogilvie J. P. (2018). Proc. Natl. Acad. Sci. U. S. A..

[cit44] Huppman P., Arlt T., Penzkofer H., Schmidt S., Bibikova M., Dohse B., Oesterhelt D., Wachtveit J., Zinth W. (2002). Biophys. J..

[cit45] Ma F., Romero E., Jones M. R., Novoderezhkin V. I., van Grondelle R. (2018). J. Phys. Chem. Lett..

[cit46] Ma F., Romero E., Jones M. R., Novoderezhkin V. I., van Grondelle R. (2019). Nat. Commun..

[cit47] Finkele U., Lauterwasser C., Zinth W., Gray K. A., Oesterhelt D. (1990). Biochemistry.

[cit48] Wang H., Hao Y., Jiang Y., Lin S., Woodbury N. W. (2012). J. Phys. Chem. B.

[cit49] Kirmaier C., He C., Holten D. (2001). Biochemistry.

[cit50] Laible P. D., Hanson D. K., Buhrmaster J. C., Tira G. A., Faries K. M., Holten D., Kirmaier C. (2020). Proc. Natl. Acad. Sci. U. S. A..

[cit51] Steffen M. A., Lao K., Boxer S. G. (1994). Science.

[cit52] Treynor T. P., Yoshina-Ishii C., Boxer S. G. (2004). J. Phys. Chem. B.

[cit53] Saggu M., Fried S. D., Boxer S. G. (2019). J. Phys. Chem. B.

[cit54] Rigby S. E. J., Nugent J. H. A., O'Malley P. J. (1994). Biochemistry.

[cit55] Diner B. A., Schlodder E., Nixon P. J., Coleman W. J., Rappaport F., Lavergne J., Vermaas W. F. J., Chisholm D. A. (2001). Biochemistry.

[cit56] Okubo T., Tomo T., Sugiura M., Noguchi T. (2007). Biochemistry.

[cit57] Saito K., Ishida T., Sugiura M., Kawakami K., Umena Y., Kamiya N., Shen J. R., Ishikita H. (2011). J. Am. Chem. Soc..

[cit58] Ishikita H., Saenger W., Biesiadka J., Loll B., Knapp E.-W. (2006). Proc. Natl. Acad. Sci. U. S. A..

[cit59] Beck M. H., Jäckle A., Worth G. A., Meyer H. D. (2000). Phys. Rep..

[cit60] Tamura H., Bittner E. R., Burghardt I. (2007). J. Chem. Phys..

[cit61] Tamura H., Ramon J. G. S., Bittner E. R., Burghardt I. (2008). Phys. Rev. Lett..

[cit62] Tamura H. (2009). J. Chem. Phys..

[cit63] Thellamurege N. M., Si D., Cui F., Zhu H., Lai R., Li H. (2013). J. Comput. Chem..

[cit64] Thellamurege N. M., Li H. (2012). J. Chem. Phys..

[cit65] Schmidt M. W., Baldridge K. K., Boatz J. A., Elbert S. T., Gordon M. S., Jensen J. H., Koseki S., Matsunaga N., Nguyen K. A., Su S., Windus T. L., Dupuis M., Montgomery Jr J. A. (1993). J. Comput. Chem..

[cit66] Yanai T., Tew D. P., Handy N. C. (2004). Chem. Phys. Lett..

[cit67] Cupellini L., Caprasecca S., Guido C. A., Müh F., Renger T., Mennucci B. (2018). J. Phys. Chem. Lett..

[cit68] Cieplak P., Caldwell J., Kollman P. (2001). J. Comput. Chem..

[cit69] Saito K., Ishikita H. (2011). Biophys. J..

[cit70] Ullmann G. M., Knapp E.-W. (1999). Eur. Biophys. J..

[cit71] Schutz C. N., Warshel A. (2001). Proteins.

[cit72] Warshel A., Sharma P. K., Kato M., Parson W. W. (2006). Biochim. Biophys. Acta.

[cit73] Umena Y., Kawakami K., Shen J.-R., Kamiya N. (2011). Nature.

[cit74] Roszak A. W., Moulisová V., Reksodipuro A. D. P., Gardiner A. T., Fujii R., Hashimoto H., Isaacs N. W., Cogdell R. J. (2012). Biochem. J..

[cit75] Grimme S. (2006). J. Comput. Chem..

[cit76] Tamura H. (2016). J. Phys. Chem. A.

[cit77] Mitsuhashi K., Tamura H., Saito K., Ishikita H. (2021). J. Phys. Chem. B.

[cit78] Kern J., Chatterjee R., Young I. D., Fuller F. D., Lassalle L., Ibrahim M., Gul S., Fransson T., Brewster A. S., Alonso-Mori R., Hussein R., Zhang M., Douthit L., de Lichtenberg C., Cheah M. H., Shevela D., Wersig J., Seuffert I., Sokaras D., Pastor E., Weninger C., Kroll T., Sierra R. G., Aller P., Butryn A., Orville A. M., Liang M., Batyuk A., Koglin J. E., Carbajo S., Boutet S., Moriarty N. W., Holton J. M., Dobbek H., Adams P. D., Bergmann U., Sauter N. K., Zouni A., Messinger J., Yano J., Yachandra V. K. (2018). Nature.

