# Supplementary material for: The origin of unidirectional charge separation in photosynthetic reaction centers: nonadiabatic quantum dynamics of exciton and charge in pigment–protein complexes
Source: Chem Sci. 2021 May 5;12(23):8131–40. doi: 10.1039/d1sc01497h (PMC8208306; doi:10.1039/d1sc01497h)
Supplement: SC-012-D1SC01497H-s001 [file SC-012-D1SC01497H-s001.pdf]

Electronic Supplementary Material (ESI) for Chemical Science.
This journal is © The Royal Society of Chemistry 2021

SI for "Origin of Unidirectional Charge Separation in Photosynthetic Reaction Centers: Nonadiabatic Quantum Dynamics of Exciton and Charge in the Pigment-Protein Complexes"

Hiroyuki Tamura, Keisuke Saito, and Hiroshi Ishikita

1. Effect of steric hindrance on the geometry of the vinyl group of P_{D1} and P_{D2}

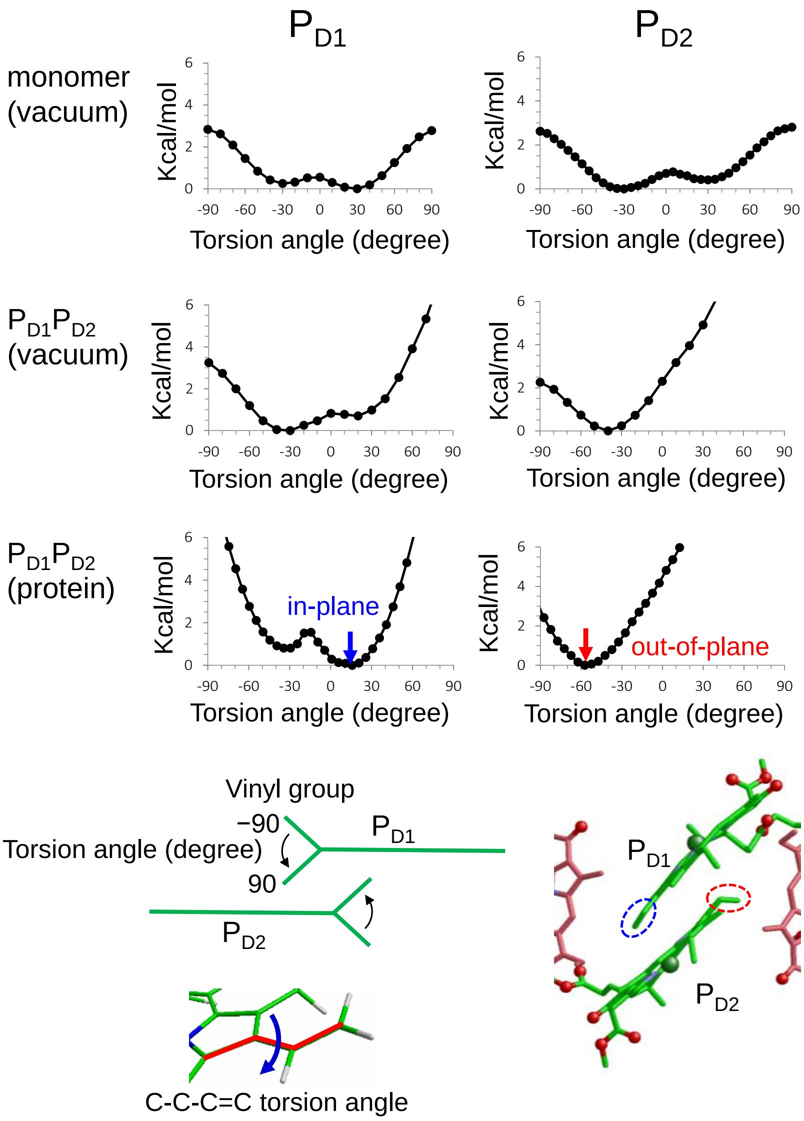

Fig. S1. Potential energy curve as a function of the C-C-C=C torsion angle of the vinyl group of P_{D1} and P_{D2} for isolated monomer, dimer in vacuum, and dimer in the protein environment of PSII. In PSII, the vinyl group of P_{D1} (marked with the blue circle) take an in-plane structure at the equilibrium geometry in the protein environment. The in-plane structure of the P_{D1} vinyl group leads to a small $\pi-\pi$ interaction with Chl_{D2}.

2. Reorganization energies of pigments

Table S1. Intramolecular reorganization energy (meV) relevant with the charge separation in PbRC. The geometries of excited states, cations, and anions are optimized from the ground state geometry, using QM/MM.

	Exciton	Cation	Anion
P_LP_M	108	95	–
B_L	24	107	140
B_M	30	143	136
H_L	103	–	180
H_M	98	–	170

Total reorganization energy (meV) of charge separated states from the ground state geometry.

$(P_LP_M)^{\bullet+}B_L^{\bullet-}$	235
$(P_LP_M)^{\bullet+}H_L^{\bullet-}$	275
$(P_LP_M)^{\bullet+}B_M^{\bullet-}$	231
$(P_LP_M)^{\bullet+}H_M^{\bullet-}$	265

Table S2. Intramolecular reorganization energy (meV) relevant with the charge separation in PSII. The geometries of excited states, cations, and anions are optimized from the ground state geometry, using QM/MM.

	Exciton	Cation	Anion
P_{D1}	32	94	–
P_{D2}	48	111	–
Chl_{D1}	47	124	165
Chl_{D2}	59	154	180
$Pheo_{D1}$	70	–	132
$Pheo_{D2}$	76	–	150

Total reorganization energy (meV) of charge separated states from the ground state geometry.

$Chl_{D1}^{\bullet+}Pheo_{D1}^{\bullet-}$	256
$P_{D1}^{\bullet+}Pheo_{D1}^{\bullet-}$	226
$P_{D1}^{\bullet+}Chl_{D1}^{\bullet-}$	259
$P_{D2}^{\bullet+}Chl_{D1}^{\bullet-}$	276
$Chl_{D2}^{\bullet+}Pheo_{D2}^{\bullet-}$	304
$P_{D2}^{\bullet+}Chl_{D2}^{\bullet-}$	291
$P_{D1}^{\bullet+}Chl_{D2}^{\bullet-}$	274
$P_{D1}^{\bullet+}Pheo_{D2}^{\bullet-}$	244

3. Delocalized exciton states in the reaction center of PSII

Delocalized exciton states in the reaction center of PSII are analyzed by diagonalizing the Hamiltonian matrix consisting of the excitation energies of pigments and excitonic couplings (Table S3). The lowest and second lowest exciton states are localized on Chl_{D1} and Chl_{D2}, respectively (Fig. S2). Thus, charge separation is considered to occur from the localized exciton.

The exciton energies calculated using TDDFT-QM/MM/PCM with the CAMB3LYP functional (μ = 0.14, α = 0.19, and β = 0.46) are systematically blue-shifted as compared to the experimental values, where the calculated lowest excitation energy in the reaction center of PSII (632 nm) is blue-shifted from the experimental value of 680 nm.

Table S3. Hamiltonian matrix consisting of the excitation energies of pigments and excitonic couplings.

	$Pheo_{D1}^*$	Chl_{D1}^*	P_{D1}^*	P_{D2}^*	Chl_{D2}^*	$Pheo_{D2}^*$
$Pheo_{D1}^*$	2048	-11	0	0	0	0
Chl_{D1}^*		1965	7	-14	0	0
P_{D1}^*			2032	-10	-14	0
P_{D2}^*				2038	7	0
Chl_{D2}^*					1992	-13
$Pheo_{D2}^*$						2044

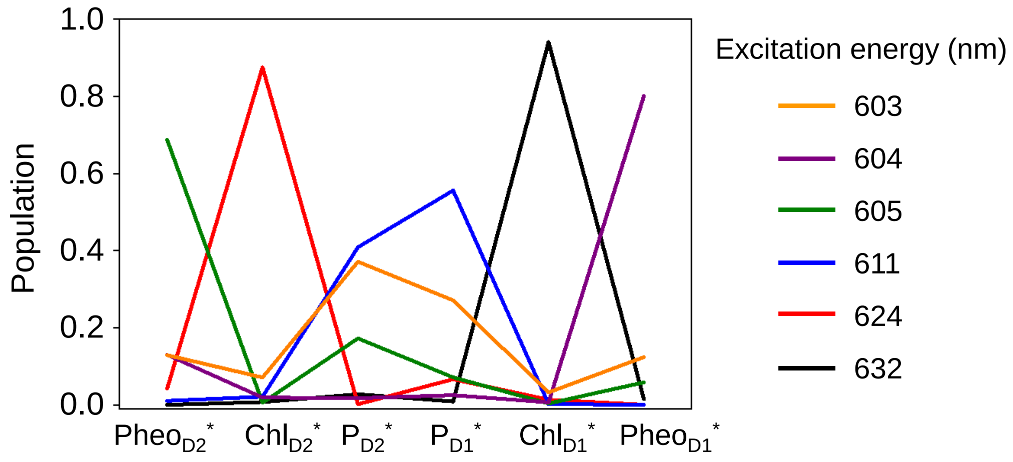

Fig. S2. Distribution of exciton on each pigment in the diagonalized (delocalized) exciton states in the reaction center of PSII.

4. Time constants based on Marcus theory

For comparison, we estimate the charge transfer rates along the active branches of PbRC and PSII using the Marcus theory. The time constants of $(P_LP_M)^*\rightarrow(P_LP_M)^{\bullet+}B_L^{\bullet-}$ and $B_L^{\bullet-}\rightarrow H_L^{\bullet-}$ transfers in PbRC are ~4 and ~0.1 ps, respectively, and those of $\mathrm{Chl}_{D1}^*\rightarrow\mathrm{Chl}_{D1}^{\bullet+}\mathrm{Pheo}_{D1}^{\bullet-}$ and $\mathrm{Chl}_{D1}^{\bullet+}\rightarrow P_{D1}^{\bullet+}$ transfers in PSII are <0.1 and ~3 ps, respectively. The time constant of $B_L^{\bullet-}\rightarrow H_L^{\bullet-}$ transfer estimated by the Marcus theory is underestimated as compared to that calculated by the quantum dynamics calculations.

5. Nonadiabatic Quantum Dynamics Calculations with the MCTDH method

Nonadiabatic quantum dynamics calculations with the multi-configuration time-dependent Hartree (MCTDH) method is briefly introduced below. In the MCTDH method [S1], multi-dimensional vibrational wavefunctions on the respective electronic states are described as a linear combination of Hartree products of single particle functions (SPFs), where the respective SPFs are described by the discrete variable representation (DVR). The vibrational wave packets on the potential energy surfaces of the respective electronic states are propagated according to the time-dependent Schrödinger equation, where both the expansion coefficients of the respective configurations and the SPFs themselves are time-dependent. The MCTDH method enables efficient multi-states nonadiabatic quantum dynamics calculations, properly considering correlations among the nuclear degrees of freedom, the Franck-Condon factor of vibrational wavefunctions (Fig. S3), and vibrational energy redistribution along with electronic state transitions. This is well suited for describing charge and exciton transfers [S2], as well as superexchange mechanisms [S3] in condensed molecular systems beyond perturbative approaches.

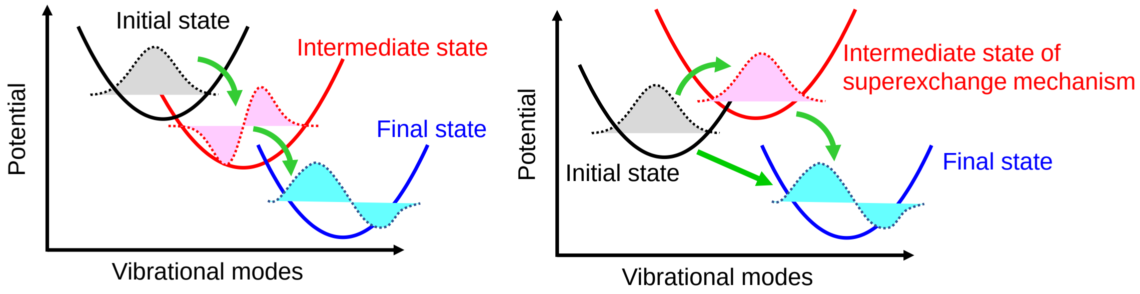

Fig. S3. Schematic illustration of vibrational wavefunctions along with electronic state transitions.

[S1] H. D. Meyer, U. Manthe, L. S. Cederbaum, *Chem. Phys. Lett.* 1990**, 165**, 73-78.; M. H. Beck, A. Jäckle, G. A. Worth and H. D. Meyer, *Phys. Rep.*, 2000, **324**, 1-105.

[S2] H. Tamura and I. Burghardt, *J Am Chem Soc.*, 2013, **135**, 16364-16367.

[S3] H. Tamura, M. Huix-Rotllant, I. Burghardt, Y. Olivier and D. Beljonne, *Phys. Rev. Lett.*, 2015, **115**, 107401-1-5.

6. Effective mode representation of linear vibronic coupling model.

We consider the following linear vibronic coupling Hamiltonian for the nonadiabatic quantum dynamics calculations:

$$H = \sum_{I} h_I(\mathbf{x}) |I\rangle\langle I| + \sum_{I \neq J} H_{IJ} \left(|I\rangle\langle J| + |J\rangle\langle I| \right) \tag{S1}$$

$$h_I(\mathbf{x}) = \sum_{i=1}^{N} \frac{\omega_i}{2} \left(p_i^2 + x_i^2 \right) + \sum_{i=1}^{N} \kappa_i^I x_i + H_{II} . \tag{S2}$$

N is the number of vibrational modes, H_{IJ} is the diabatic coupling (electronic coupling) between the state I and J. H_{II} is the vertical excitation energy of the Ith electronic states. ω_i, x_i, and p_i are the frequency, position, and momentum of the ith normal mode in the dimensionless coordinate. κ_i^I is the vibronic coupling of the ith normal mode in the Ith electronic state. The linear vibronic coupling model (Eq. S2) can be transformed to another equivalent representation (Eq. S3) via an orthogonal transformation of the coordinates, $\boldsymbol{X} = \boldsymbol{U}\,\boldsymbol{x}$, from the normal modes, $\boldsymbol{x}$, to the hierarchical electron-phonon model as follows [S4-S6] (for a two-state model):

$$h_I(\mathbf{x}) = \sum_{i=1}^{N} \frac{\Omega_i}{2} \left(P_i^2 + X_i^2 \right) + D_1 X_1 + \sum_{i=1}^{N} d_{i,i+1} \left(P_i P_{i+1} + X_i X_{i+1} \right) + H_{II} . \tag{S3}$$

The frequencies, $\Omega_{\rm i}$, and bilinear couplings, $d_{\rm ij}$, are expressed with the elements, $u_{\rm ij}$, of the orthogonal matrix, $\boldsymbol{U}$:

$$\Omega_i = \sum_{j=1}^{N} \omega_j u_{ij}^2 , \quad d_{ij} = \sum_{k=1}^{N} \omega_k u_{ik} u_{jk} . \tag{S4}$$

The cumulative effect of the vibronic couplings of all normal modes is represented by a single effective mode, X_1, with the effective vibronic coupling, D_1:

$$D_1 X_1 = \sum_{j=1}^{N} \kappa_i^I x_i \tag{S5}$$

The transformed vibrational modes comprise a chain of coupled oscillators from X_1 to $X_{\rm N}$ via $d_{\rm ij}$ (Eq. S3). Note that Eqs. S2 and S3 are equivalent when all modes (N modes) are considered. It has been proven that the nonadiabatic dynamics considering N modes can be reproduced for a certain

propagation time by considering M effective modes ($M < N$) via truncation of the chain-like coupled oscillators of Eq. S3 [S4]. This feature can be exploited to construct a reduced model for analyzing short-time dynamics, where quantitative accuracy can be improved systematically by increasing the number of effective modes according to the order of chain. When the vibrational modes are coupled to several electronic states simultaneously, several effective vibronic coupling terms, $D_i X_i$, are needed as detailed in Refs. S4. The reorganization energy can also be reproduced by considering adequate number of effective modes. In the present study, the effective modes, X_i, for each pigment are determined via orthogonal transformation of the intramolecular normal modes, x_i, considering the vibronic coupling, κ_i^I, for the exciton, cation, and anion states.

[S4] H. Tamura, E. R. Bittner and I. Burghardt, *J. Chem. Phys.*, 2007, **127**, 034706.

[S5] H. Tamura, J. G. S. Ramon, E. R. Bittner and I. Burghardt, *Phys. Rev. Lett.*, 2008, **100**, 107402.

[S6] H. Tamura, *J. Chem. Phys.*, 2009, **130**, 214705.
