# Supplementary material for: The origin of unidirectional charge separation in photosynthetic reaction centers: nonadiabatic quantum dynamics of exciton and charge in pigment–protein complexes
Source: Chem Sci. 2021 May 5;12(23):8131–40. doi: 10.1039/d1sc01497h (PMC8208306; doi:10.1039/d1sc01497h)
Supplement: SC-012-D1SC01497H-s002 [file SC-012-D1SC01497H-s002.pdf]

Electronic Supplementary Material (ESI) for Chemical Science.
This journal is © The Royal Society of Chemistry 2021

SI for "Origin of Unidirectional Charge Separation in Photosynthetic Reaction Centers: Nonadiabatic Quantum Dynamics of Exciton and Charge in the Pigment-Protein Complexes"

The paramters for the vibrational modes of pigments in the nonadiabatic quantum dynamics calculations, i.e., the frequency, ω, and the linear vibronic coupling, κ, in the following equation are summzrized below (in eV).

$$h_I(\mathbf{x}) = \sum_i \frac{\omega_i}{2}\left(x_i^2 + p_i^2\right) + \sum_i \kappa_i^I x_i + H_{II}$$

ω_i, x_i, and p_i are the frequency, position, and momentum of the ith vibrational mode. κ_i^I is the vibronic coupling of the ith vibrational mode in the Ith electronic state.

==================================

Charge Separation in Purple Bacterial Reaction Center

ω_i for (P_LP_M) special pair

w1 = 0.456665
w2 = 0.456552
w3 = 0.435041
w4 = 0.418677
w5 = 0.396689
w6 = 0.391493
w7 = 0.367777
w8 = 0.360828
w9 = 0.310860
w10 = 0.306468
w11 = 0.270594
w12 = 0.230775
w13 = 0.205526
w14 = 0.189664
w15 = 0.173582
w16 = 0.147218
w17 = 0.129304
w18 = 0.103937
w19 = 0.091683
w20 = 0.067871
w21 = 0.047938
w22 = 0.028602
w23 = 0.022375
w24 = 0.013945
w25 = 0.009199

κ_i for an exciton on the (P_LP_M) special pair

k1 = 0.003483
k2 = 0.002648
k3 = -0.007756
k4 = -0.001183
k5 = 0.016766
k6 = 0.010517
k7 = 0.081278
k8 = -0.005178
k9 = -0.003229
k10 = 0.008399
k11 = 0.040045
k12 = -0.008091
k13 = -0.085594
k14 = 0.022482
k15 = -0.090752
k16 = 0.022279
k17 = 0.055648
k18 = -0.005350
k19 = -0.040382
k20 = -0.006683
k21 = -0.020467
k22 = -0.007660
k23 = 0.017525
k24 = 0.007628
k25 = -0.011194

κ_i for a hole on the (P_LP_M) special pair

k1 = 0.002023
k2 = 0.008927
k3 = 0.008691
k4 = -0.006803
k5 = -0.003074
k6 = 0.028962
k7 = 0.031987
k8 = 0.015664
k9 = 0.017663
k10 = -0.006522
k11 = -0.013098
k12 = -0.042542
k13 = -0.010233
k14 = 0.092059
k15 = -0.023651
k16 = 0.089663
k17 = 0.001007
k18 = -0.056907

k19 = -0.005113
k20 = -0.029656
k21 = -0.003865
k22 = -0.022719
k23 = -0.004711
k24 = 0.019485
k25 = 0.001691

ω_i for B_L and B_M
w1 = 0.466550
w2 = 0.453095
w3 = 0.444364
w4 = 0.436117
w5 = 0.418372
w6 = 0.398127
w7 = 0.388133
w8 = 0.381088
w9 = 0.335934
w10 = 0.268831
w11 = 0.238658
w12 = 0.222495
w13 = 0.204893
w14 = 0.186229
w15 = 0.168951
w16 = 0.138927
w17 = 0.121557
w18 = 0.097984
w19 = 0.082673
w20 = 0.059428
w21 = 0.043969
w22 = 0.030957
w23 = 0.020427
w24 = 0.012257
w25 = 0.008223

κ_i for an electron on B_L and B_M
k1 = 0.014938
k2 = 0.004291
k3 = 0.019477
k4 = 0.003240
k5 = 0.008527
k6 = -0.008880
k7 = 0.023322
k8 = -0.022010
k9 = -0.010981
k10 = 0.044365

k11 = -0.025966
k12 = 0.058101
k13 = 0.106698
k14 = -0.083843
k15 = 0.079829
k16 = -0.052670
k17 = -0.050604
k18 = -0.058731
k19 = 0.031184
k20 = -0.016328
k21 = -0.020984
k22 = 0.016248
k23 = -0.013256
k24 = -0.007446
k25 = -0.001703

ω_i for H_L and H_M
w1 = 0.445660
w2 = 0.433760
w3 = 0.406192
w4 = 0.402452
w5 = 0.393309
w6 = 0.384571
w7 = 0.378176
w8 = 0.364391
w9 = 0.287780
w10 = 0.222896
w11 = 0.213305
w12 = 0.202523
w13 = 0.184927
w14 = 0.169664
w15 = 0.147163
w16 = 0.128129
w17 = 0.110591
w18 = 0.088544
w19 = 0.074124
w20 = 0.055158
w21 = 0.039458
w22 = 0.029678
w23 = 0.018266
w24 = 0.010776
w25 = 0.006627

κ_i for an electron on H_L and H_M
k1 = 0.010597
k2 = -0.005122

k3 = -0.049950
k4 = -0.008288
k5 = -0.013771
k6 = -0.014190
k7 = -0.012133
k8 = -0.002712
k9 = 0.000011
k10 = -0.030611
k11 = 0.093988
k12 = -0.088225
k13 = 0.091404
k14 = 0.074692
k15 = 0.081364
k16 = 0.061694
k17 = -0.051234
k18 = 0.056532
k19 = 0.034264
k20 = -0.019669
k21 = -0.025852
k22 = 0.018031
k23 = 0.012311
k24 = 0.006028
k25 = 0.004436

==================================

Charge Separation along the D1-Branch in Photosystem II

ω_i for Chl_{D1}
w1 = 0.466416
w2 = 0.466178
w3 = 0.449356
w4 = 0.429996
w5 = 0.414661
w6 = 0.396472
w7 = 0.386146
w8 = 0.379171
w9 = 0.345207
w10 = 0.260024
w11 = 0.212830
w12 = 0.207285
w13 = 0.190702
w14 = 0.181014
w15 = 0.160231
w16 = 0.143206

w17 = 0.127042
w18 = 0.099619
w19 = 0.090772
w20 = 0.061947
w21 = 0.057515
w22 = 0.034035
w23 = 0.031673
w24 = 0.014461
w25 = 0.011622

κ_i for an exciton on Chl_{D1}
k1 = 0.004556
k2 = -0.008440
k3 = 0.001429
k4 = -0.001527
k5 = -0.000458
k6 = 0.008118
k7 = -0.002258
k8 = -0.007274
k9 = 0.000907
k10 = -0.000309
k11 = 0.024379
k12 = 0.036338
k13 = 0.062078
k14 = 0.007892
k15 = -0.079788
k16 = 0.012623
k17 = 0.044638
k18 = 0.000018
k19 = -0.026496
k20 = 0.002882
k21 = -0.017410
k22 = -0.010967
k23 = 0.000748
k24 = -0.002657
k25 = 0.001276

κ_i for a hole on Chl_{D1}
k1 = 0.029548
k2 = 0.031546
k3 = -0.056932
k4 = 0.000867
k5 = -0.022714
k6 = 0.006662
k7 = -0.018219
k8 = 0.002338
k9 = -0.001016

k10 = -0.004759
k11 = -0.062120
k12 = 0.047298
k13 = 0.031685
k14 = 0.103938
k15 = -0.065488
k16 = 0.081568
k17 = 0.024429
k18 = 0.051135
k19 = -0.004484
k20 = 0.035136
k21 = -0.002643
k22 = -0.004312
k23 = -0.025954
k24 = 0.002943
k25 = 0.008754

ω_i for P_{D1}
w1 = 0.452609
w2 = 0.439089
w3 = 0.406988
w4 = 0.403767
w5 = 0.393584
w6 = 0.388311
w7 = 0.378154
w8 = 0.374912
w9 = 0.241293
w10 = 0.221903
w11 = 0.201736
w12 = 0.197731
w13 = 0.177892
w14 = 0.168480
w15 = 0.151672
w16 = 0.134259
w17 = 0.115341
w18 = 0.094415
w19 = 0.086410
w20 = 0.053291
w21 = 0.050067
w22 = 0.030222
w23 = 0.026889
w24 = 0.016161
w25 = 0.011622

κ_i for a hole on P_{D1}
k1 = 0.005684

k2 = 0.015802
k3 = -0.018143
k4 = 0.001962
k5 = 0.007403
k6 = 0.011369
k7 = 0.003912
k8 = 0.011787
k9 = -0.002128
k10 = -0.037253
k11 = 0.010880
k12 = -0.085149
k13 = -0.026034
k14 = -0.097229
k15 = -0.059351
k16 = -0.060356
k17 = 0.029951
k18 = -0.038131
k19 = -0.020855
k20 = 0.023242
k21 = -0.010674
k22 = 0.016428
k23 = 0.006735
k24 = 0.009863
k25 = -0.003061

ω_i for $Pheo_{D1}$
w1 = 0.456138
w2 = 0.448054
w3 = 0.443815
w4 = 0.432901
w5 = 0.401064
w6 = 0.393183
w7 = 0.383318
w8 = 0.371525
w9 = 0.362734
w10 = 0.315993
w11 = 0.222199
w12 = 0.209630
w13 = 0.197064
w14 = 0.178983
w15 = 0.155604
w16 = 0.138768
w17 = 0.117955
w18 = 0.095185
w19 = 0.080595
w20 = 0.059815

w21 = 0.044245
w22 = 0.031087
w23 = 0.019590
w24 = 0.014566
w25 = 0.007429

κ_i for an electron om $Pheo_{D1}$
k1 = 0.001257
k2 = 0.001394
k3 = -0.011054
k4 = 0.019760
k5 = 0.008092
k6 = 0.018595
k7 = -0.020096
k8 = 0.012760
k9 = 0.006095
k10 = 0.000048
k11 = -0.060882
k12 = 0.081701
k13 = -0.088696
k14 = -0.075494
k15 = 0.062045
k16 = -0.070605
k17 = 0.040130
k18 = 0.047032
k19 = -0.033883
k20 = 0.018162
k21 = 0.023284
k22 = 0.021144
k23 = -0.010986
k24 = -0.007462
k25 = -0.002672

===================================

For the exciton transfer from Chl_{D2} to Chl_{D1} via P_{D1} and P_{D2}, the vibronic parameters for Chl_{D2} are identical with those of Chl_{D1}, and 10 effective vibrational modes are considered for the intermediate exciton states on P_{D1} and P_{D2} as follows.

ω_i for P_{D1} and P_{D2}
w1 = 0.451080
w2 = 0.412854
w3 = 0.386839
w4 = 0.338898
w5 = 0.201736
w6 = 0.166850

w7 = 0.129492
w8 = 0.088103
w9 = 0.043040
w10 = 0.016315

κ_i for an exciton on P_{D1}
kd1 = 0.002888
kd2 = -0.004957
kd3 = 0.010324
kd4 = -0.002066
kd5 = -0.051456
kd6 = 0.069345
kd7 = -0.049491
kd8 = 0.034079
kd9 = -0.015433
kd10 = -0.006541

κ_i for an exciton on P_{D2}
kx1 = 0.003114
kx2 = -0.005345
kx3 = 0.011132
kx4 = -0.002228
kx5 = -0.055485
kx6 = 0.074774
kx7 = -0.053366
kx8 = 0.036747
kx9 = -0.016641
kx10 = -0.007053
